# Effect of combination sildenafil and gemfibrozil on cisplatin-induced nephrotoxicity; role of heme oxygenase-1

**DOI:** 10.1080/0886022X.2018.1455596

**Published:** 2018-04-30

**Authors:** Safaa Behiry, Ahmed Rabie, Mahmoud Kora, Wesam Ismail, Dina Sabry, Ahmed Zahran

**Affiliations:** a Department of Physiology, Faculty of Medicine, Cairo University, Cairo, Egypt;; b Department of Internal Medicine, Faculty of Medicine, Menoufia University, Shebeen El-Kom, Egypt;; c Department of Pathology, Faculty of Medicine, Beni Suef University, Beni Suef, Egypt;; d Department of Biochemistry, Faculty of Medicine, Cairo University, Cairo, Egypt

**Keywords:** Acute kidney injury, cisplatin nephrotoxicity, gemfibrozil, heme oxygenase-1, oxidative stress and sildenafil

## Abstract

**Background/aim:** Cisplatin-induced nephrotoxicity in large proportion of patients. The aim of this work is to clarify the effect of combination of sildenafil and gemfibrozil on cisplatin-induced nephrotoxicity either before or after cisplatin treatment and determination of nephrotoxicity predictors among the measured tissue markers.

**Methods:** Thirty two adult male albino rats were divided into four equal groups (G) GI control, GII received cisplatin, GIII received sildenafil and gemfibrozil before cisplatin, GIV received sildenafil and gemfibrozil after cisplatin. Creatinine and urea were measured and animals were sacrificed and kidney was taken for histopathology. The following tissue markers were measured, heme oxygenase-1 (HO-1) activity, reduced glutathione, quantitative (real-time polymerase chain reaction) RT-PCR for gene expression of tumor necrosis factor alpha (TNF-α) and endothelial nitric oxide synthase (ENOS) level.

**Results:** GII developed AKI demonstrated by significantly high urea and creatinine and severe diffuse (80–90%) tubular necrosis. TNF-α was highly and significantly elevated while the rest of tissue markers were significantly reduced in GI1 compared to other groups. GIV showed better results compared to GIII. There was a significant positive correlation between creatinine and TNF-α when combining GI and GII while there were significant negative correlation between creatinine and other tissue markers in same groups. Linear regression analysis demonstrated that HO-1 was the independent predictor of AKI demonstrated by elevated creatinine among GI and GII.

**Conclusions:** Combination of sildenafil and gemfibrozil can be used in treatment of cisplatin-induced nephrotoxicity. HO-1 is a promising target for prevention and/or treatment of cisplatin-induced nephrotoxicity.

## Introduction

Cisplatin is a chemotherapeutic agent used in treatment of different types of cancers; however, its use is limited due to its nephrotoxicity [1]. More than 25% of patients develop acute nephrotoxicity after receiving cisplatin, due to its accumulation within the proximal tubular cells of the kidney [2]. Cisplatin activates apoptotic pathway and inflicts cellular damage via oxidative stress and inflammation [3]. Tumor necrosis factor alpha (TNF-α) receptors were found to be highly upregulated in the kidney and participate in both necrosis and apoptosis of renal epithelial cells in cisplatin nephrotoxicity [4]. HO-1, the rate-limiting enzyme in the catabolism of heme was found to be protective against cisplatin nephrotoxicity through inhibition of inflammation, oxidative stress, and apoptosis [5,6].

Many studies demonstrated reduction of ENOS and reduced glutathione in cisplatin-induced nephrotoxicity [7,8].

Sildenafil, a phosphodiesterase 5 (PDE5) inhibitor was shown to ameliorate oxidative stress and DNA damage in mice with renovascular hypertension [9] and prevent sepsis-induced AKI through maintenance of oxidant/antioxidant status and reduction of level of TNF-α [10]. Also sildenafil was shown to attenuate renal injury induced by cisplatin nephrotoxicity [11].

Gemfibrozil is a fibrate medication considered as one of peroxisome proliferator-activated receptors alpha (PPAR-α) agonists used to lower triglycerides and cholesterol [12]. PPAR agonist showed positive regulation of ENOS with beneficial effect on cardiovascular disease [13]. PPAR-activation by caloric restriction was shown to protect against cisplatin-induced nephrotoxicity [14] and PPAR-α ligands confirmed a protective effect against cisplatin-mediated renal proximal tubules necrosis [15]. Fibrates inhibits apoptotic cell death and considered a promising agent to ameliorate renal proximal tubular cell death in cisplatin treatment [16,17].

The aim of this work is to clarify the effect of sildenafil and gemfibrozil in combination on cisplatin-induced nephrotoxicity either before cisplatin treatment as a prophylactic measure or postcisplatin treatment as a therapeutic measure with determination of nephrotoxicity predictors among studied markers.

## Materials and methods

Thirty two adult male albino rats, weighing 200–250 g were obtained from the animal house of Faculty of Medicine, Cairo University, Egypt. The animals were housed in wire mesh cages at room temperature with day light–dark cycles and maintained on standard rat chow and tap water. Veterinary care was provided by Animal House Unit of Faculty of Medicine, Cairo University. This study was approved by ethical Committee of Faculty of Medicine, Cairo and Menoufia Universities.

Thirty two adult male albino rats were divided into four groups of eight rats each (see Table 1 for time table treatment).

**Table 1.** Time table for treatment of all groups.

GI	Days 1–14 Usual diet	Day 15 IP saline	Day 17 Sacrifice
GII	Days 1–14 Usual diet	Day 15 IP cisplatin	Day 17 Sacrifice
GIII	Days 1–14 Sildenafil and gemfibrozil treatment	Day 15 IP cisplatin	Day 17 Sacrifice
GIV	Day 1 IP cisplatin	Days 3–16 Sildenafil and gemfibrozil treatment	Day 17 Sacrifice

Group I (*n* = 8): Control group, received usual diet for 14 days then intraperitoneal injection of normal saline 2 mL/kg.

Group II (*n* = 8): Cisplatin-induced nephrotoxicity group, given usual diet for 14 days then received cisplatin intraperitoneally in a dose of 5 mg/kg body weight (single dose).

Group III (*n* = 8): Combined sildenafil and gemfibrozil treated before cisplatin-induced nephrotoxicity, received sildenafil orally in a dose of 40 mg/kg body weight once daily concomitant with gemfibrozil orally in a dose of 100 mg/kg body weight once daily for 14 days then received cisplatin intraperitoneally in a dose of 5 mg/kg bodyweight (single dose).

Group IV (*n* = 8): Combined sildenafil and gemfibrozil-treated after cisplatin-induced nephrotoxicity, received cisplatin intraperitoneally in a dose of 5 mg/kg bodyweight (single dose), then received sildenafil orally in a dose of 40 mg/kg body weight once daily concomitant with gemfibrozil orally in a dose of 100 mg/kg body weight once daily for 14 days.

### Feasibility study was done on nine rats

Three rats were treated with cisplatin and kept on usual diet and left for possibility of spontaneous recovery; however, all of them died within 2 weeks.

Three rats were treated with combination of sildenafil and gemfibrozil only and did not show any significant difference with control group.

Three rats were treated with cisplatin only and sacrificed after 48 h. Urea and creatinine were elevated and kidney biopsy showed severe tubular necrosis confirming that cisplatin nephrotoxicity occurs within 48 h.

### Drugs

Cisplatin vial 10 mg/10 mL (Merck, Kenilworth, NJ, USA) and was administered intraperitoneally in a dose of 5 mg/kg body weight, prepared as 10 mg/mL in 0.5% carboxy methyl cellulose solution, between 8:00 am and 10:00 am.

Sildenafil treatment was performed with Viagra (orally in a dose of 40 mg/kg body weight once daily; Pfizer Australia Pty Ltd., West Ryde, Australia), gemfibrozil tablets (Lopid, Alkan pharma) were administered orally in a dose of 100 mg/kg body weight, prepared as 10 mg/mL in 0.5% carboxy methyl cellulose solution. Oral treatment was given through gavage. Vehicle was given to control group.

The animals were fasted and deprived of water overnight (10–12 h) prior to anesthesia and sacrifice. At the end of the experimental period (day 17), blood samples were obtained for chemical analysis. Rats were anesthetized by intraperitoneal administration of a mix of ketamine (50 mg mL^−1^, 10 mg kg^−1^, Ketalar; Bayer, Leverkusen, Germany) and xylazine (2%, 0.1 mL kg^−1^; Rompun; Bayer, Leverkusen, Germany) in solution (2 mL kg^−1^).

### Tissue preparation

The left kidney was excised immediately after anesthetization. The capsule and inner medullary area of the kidney were removed, and the kidney was then cut into three transverse sections. Two pieces were snap-frozen in liquid nitrogen and stored at −70 °C for RNA extraction and protein analysis. The other portion was fixed in 10% buffered formaldehyde at room temperature and then embedded in Paraplast (Sherwood Medical, St. Louis, MO, USA) for light microscopy.

### Examination by light microscopy

Pieces of kidney embedded in paraffin were cut into four micron sections and mounted on glass slides. The sections were then deparaffinized with xylene, counterstained routinely with hematoxylin and eosin, periodic acid-Schiff (PAS) and Masson trichrome, and examined under a light microscope (200× magnifications, Dialux 22; Leitz, Milan, Italy). We evaluated brush border loss, vacuolation, and desquamation of epithelial cells in renal tubules. Five different fields of the renal outer medullary area were examined in every slide by a renal pathologist blind to the study groups. The magnitude of damage in the tubular epithelial cells was examined in PAS stained sections by counting the number of tubules that were injured in the outer medulla.

### Biochemical assessment

Plasma urea and creatinine were measured using commercial kits purchased from Human GmbH (Wiesbaden, Germany). Urea and creatinine were measured according to Patton and Crouch [18] and Henry [19].

### Heme oxygenase-1 (HO-1) activity

HO-1 activity was measured according to Lowry et al. [20] and Abraham et al. [21]. Homogenized kidney samples were incubated with heme (50 mmol/L), rat liver cytosol (5 mg/mL), MgCl_2_ (2 mM/L), glucose-6-phosphate dehydrogenase (1 U), glucose-6-phosphate (2 mM/L), and reduced nicotinamide adenine dinucleotide phosphate (NADPH) (0.8 mM/L) in 0.5 mL of 0.1 M/L phosphate buffer saline (pH 7.4) for 60 min at 37 °C. The reaction was stopped by putting the tubes on ice, and the reaction solution was extracted with chloroform. The rate of bilirubin formation was monitored at 464 nm and 520 nm by a spectrophotometer (pmol bilirubin/mg protein).

### Reduced glutathione

The kidney samples were deproteinized with 5% 5-sulfosalicylic acid solution, centrifuged to remove the precipitated protein, and then assayed for reduced glutathione expression level [22].

### Quantitative RT-PCR [23,24]

RT-PCR was performed for quantitative genes expression of TNF-α kidney tissues of all studied groups and lysed and total RNA was isolated with Thermo Fisher Scientific Inc. (Darmstadt, Germany) (GeneJET, Kit, #K0732). Ten nanograms of the total RNA from each sample were used for reverse transcription with subsequent amplification with Bioline, a median life science company, UK (SensiFAST™ SYBR^®^ Hi-ROX, one-Step Kit, catalog no.PI-50217V) in a 48-well plate using the Step One instrument (Applied Biosystem, Foster City, CA, USA). Thermal profile was 45 °C for 15 min one cycle (for cDNA synthesis) followed by 40 cycles amplification for each cycle: 10 s at 95 °C, 30 s at 60 °C, and 30 s at 72 °C. Changes in the expression of each target gene were normalized relative to the mean critical threshold (CT) values of GAPDH as housekeeping gene by the ΔΔCt method. TNF-α primer sequence was as follows: forward: AACTCGAGTGACAAGCCCGTAG and reverse: GTACCACCAGTTGGTTGTCTTTGA (gene bank accession number: XM_008772775.1). GAPDH primer sequence was as follows: forward: CACCCTGTTGCTGTAGCCATATTC and reverse: GACATCAAGAAGGTGGTGAAGCAG (gene bank accession number: XR_598347.1).

### Measurement of endothelial nitric oxide synthase (ENOS)

Kidney tissue samples (50 mg) stored in a specific lysis buffer were homogenized and centrifuged at 600×*g* at 4 °C for 10 min. The supernatant was used for ENOS assay using the ELISA kit supplied by R&D Systems, Inc. (Minneapolis, MN, USA).

### Statistical analysis

Data were analyzed using SPSS version 22 (SPSS Inc., Chicago, IL, USA) and excel sheet. Results were expressed as mean ± standard deviation. Histopathological data were expressed as percentage. One-way ANOVA test was used to compare more than numerical variable with *post hoc* analysis was done to detect significance difference between two groups. Pearson’s correlation test was done among the control and cisplatin nephrotoxicity groups to determine the relation between tissue marker and serum creatinine. Linear regression analysis was done among control (GI) and cisplatin nephrotoxicity group (GII) to detect the independent predictor for cisplatin nephrotoxicity and done again among GII and GIV to detect the independent predictor for serum creatinine improvement. Results were considered significant if *p* values <.05.

## Results

A group of 32 adult male albino rats, weighing 200–250 g were divided into four groups to study the effect of combination of sildenafil and gemfibrozil on cisplatin-induced nephrotoxicity either before cisplatin treatment as prophylactic measure or postcisplatin treatment as therapeutic measure. Blood samples were collected and all animals were anesthetized and sacrificed for tissue analysis and kidney histopathology.

ANOVA test showed significant difference among the four studied groups. *Post hoc* analysis showed the significant difference between two groups (Table 2). GIV showed better results when compared to GIII regarding urea, creatinine, HO-1, TNFα, and histopathological changes.

**Table 2.** Laboratory data and tissue markers of all studied groups.

	Group I (no. 8) Animals treated with saline (control)	Group II (no. 8) Animals treated with cisplatin	Group III (no. 8) Animals treated with sildenafil and gemfibrozil before cisplatin	Group IV (no. 8) Animals treated with sildenafil and gemfibrozil after cisplatin	F	*p* value	Post hoc
Urea (mg/dL)	20.88 ± 4.45	64.88 ± 8.27	50.87 ± 2.47	41.87 ± 6.01	83.43	.000	All are significant
Creatinine (mg/dL)	0.53 ± 0.13	3.262 ± 0.41	1.35 ± 0.23	0.96 ± 0.24	156.48	.000	All are significant
HO-1 (pmol bilirubin/mg protein)	13.24 ± 1.60	2.96 ± 0.32	9.30 ± 1.16	13.10 ± 1.01	146.29	.000	All are significant except (I vs. IV)
Reduced glutathione (mmol/g)	1.76 ± 0.62	0.70 ± 0.21	1.95 ± 0.29	1.84 ± 0.48	14.54	.000	All are significant except (I vs. III), (I vs. IV), (III vs. IV)
TNF-α gene expression	0.35 ± 0.12	1.80 ± 0.49	0.99 ± 0.34	0.61 ± 0.22	30.39	.000	All are significant except (I vs. IV)
ENOS (pg/mL)	26.95 ± 7.26	12.29 ± 2.03	20.13 ± 3.51	24.45 ± 4.38	14.96	.000	All are significant except (I vs. IV), (III vs. IV)

HO-1: heme oxygenase-1; TNF-α: tumor necrosis factor alpha; ENOS: endothelial nitric oxide synthase.

**Figure 1.**
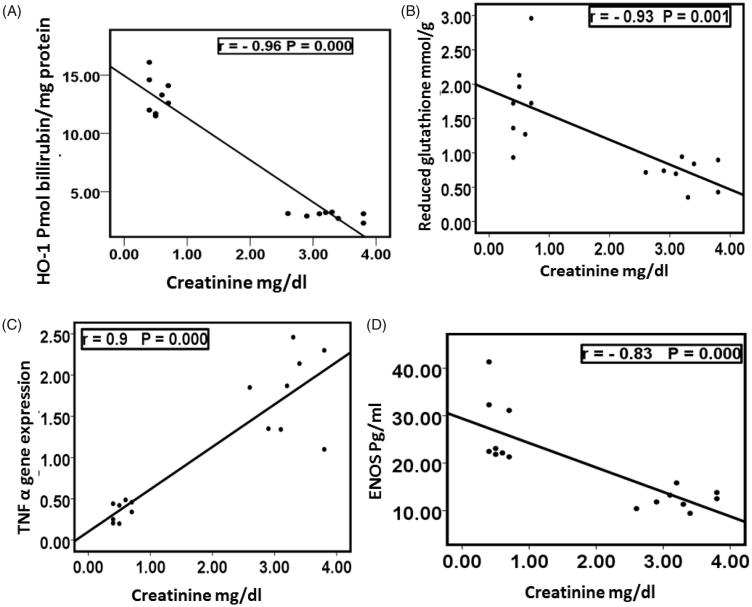
Correlation between creatinine with HO-1 (A), reduced glutathione (B), TNF α (C), and ENOS (D) in groups I and II. HO-1: heme oxygenase 1; TNF-α: tumor necrosis factor alpha; ENOS: endothelial nitric oxide synthase.

There was a significant negative correlation between creatinine with HO-1 activity, reduced glutathione and ENOS while there was a significant positive correlation between creatinine with TNFα among groups I and II (Figure 1). This showed that cisplatin nephrotoxicity detected by increased creatinine was associated with decreased HO-1 activity, reduced glutathione, and ENOS while it was associated with increased levels of TNFα.

**Figure 2.**
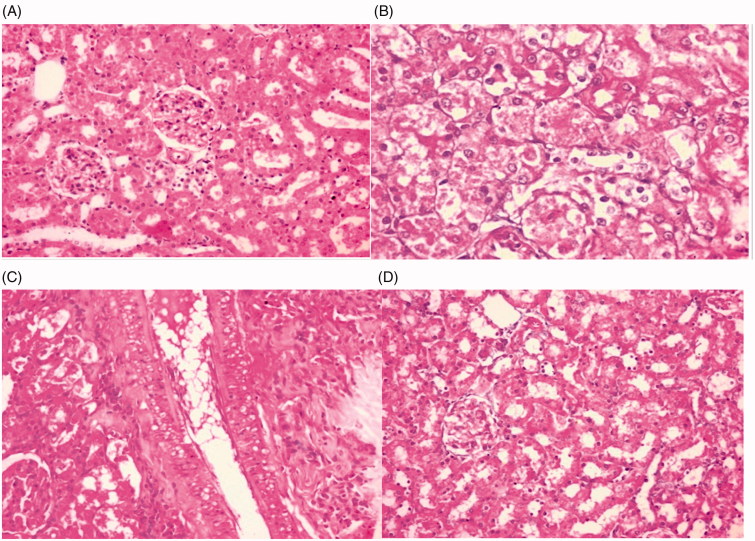
Light microscopy sections of renal tissues of rats given, stained by hematoxylin and eosin in all studies groups. (A) Group I (0–10% necrosis, no tubular atrophy). (B) Group II (80–90% necrosis, severe diffuse tubular atrophy). (C) Group III (10–20% necrosis, mild patchy tubular atrophy. (D) Group IV (0–10% necrosis, minimal tubular atrophy).

Kidney biopsies were examined for all groups to show the grades of tubular injury and percentage of necrosis (Figure 2). The pathological changes were so minimal in group IV treated with both sildenafil and gemfibrozil postcisplatin nephrotoxicity.

Linear regression analysis was done to detect the independent predictor for creatinine changes between group I and group II (Table 3). HO-1 was found to be highly and inversely associated with serum creatinine.

**Table 3.** Linear regression analysis for detecting the independent predictor for serum creatinine among control group I and group II.

Variable	B	S.E.	Beta	*p* value
HO-1	–0.2	0.05	–0.753	.002
Reduced glutathione	–0.1	0.24	–0.049	.678
TNF-α	0.32	0.31	0.175	.345
ENOS	–0.004	0.02	–0.024	.865

HO-1: heme oxygenase 1; TNF-α: tumor necrosis factor alpha; ENOS: endothelial nitric oxide synthase.

Also linear regression analysis was done between group II and group IV to detect the independent predictor for improvement of serum creatinine (Table 4). Improved HO-1 activity was found to be highly associated with improvement of serum creatinine.

**Table 4.** Linear regression analysis for detecting the independent predictor for serum creatinine among group II and group IV.

Variable	B	S.E.	Beta	*p* value
HO-1	–0.23	0.04	–1.006	.000
Reduced glutathione	0.08	0.26	0.044	.767
TNF-α	0.09	0.25	0.050	.737
ENOS	0.008	0.24	0.047	.741

HO-1: heme oxygenase 1; TNF-α: tumor necrosis factor alpha; ENOS: endothelial nitric oxide synthase.

## Discussion

Cisplatin – a chemotherapeutic agent – is associated with nephrotoxicity or acute kidney injury and occurs in about 30% of patients [25]. Many drugs were studied for possible ameliorative effect on cisplatin nephrotoxicity including sildenafil and gemfibrozil [11,26–30]. In this work, we studied the effect of sildenafil and gemfibrozil on cisplatin-induced nephrotoxicity either before cisplatin nephrotoxicity as a prophylactic measure or postcisplatin nephrotoxicity as a therapeutic measure. Cisplatin-induced severe tubular necrosis with significant deterioration of kidney function as demonstrated by elevated urea and creatinine in group treated with cisplatin compared to control group. It was reported that cisplatin is one of the drugs that induced AKI with higher odds ratio >10 [31]. Many reports of animal models demonstrated same kidney structural damage [32,33].

The mechanism of cisplatin-induced nephrotoxicity was proposed due to accumulation of cisplatin in proximal tubules with consequence depletion of antioxidants and accumulation of reactive oxygen species (ROS) and production of inflammatory markers that cause tubular damage [10]. In consistent with this, cisplatin nephrotoxicity group showed significant decrease of levels of antioxidants HO-1, reduced glutathione, and vasodilator ENOS while there was significant increase of inflammatory marker TNF α. Many studies addressed the pathophysiological mechanisms of cisplatin-induced nephrotoxicity and its targets of nephroprotection [11,17,34–39].

Combination of sildenafil and gemfibrozil was given before and after cisplatin nephrotoxicity. Pre- and post-therapy showed better results compared to cisplatin nephrotoxicity. Post-therapy showed better results compared to pretherapy with nearly complete recovery as demonstrated by histological finding. Also all tissue markers showed marked significant improvement in group IV (post-cisplatin combination therapy) compared to cisplatin nephrotoxicity group. The most important is that all tissue markers in group IV were recovered nearly normal as there was no significant difference when compared to control group. The better therapeutic effect of combination drugs can be explained by combined effect of activation of anti-oxidants and anti-inflammatory mechanisms in addition to vasodilatation mechanisms of phosphodiesterase inhibitor sildenafil and carbon monoxide, the product of heme oxygenase activity.

The better response to post-treatment can be explained by continuous activation of protective mechanisms induced by combination therapy preventing cisplatin nephrotoxicity. However, in pretreatment, protective mechanisms were activated, discontinuation of drugs leads to gradual reduction of protective mechanisms with less favorable response compared to post-treatment group.

We could not identify any article that addressed the same combination therapy either before or after cisplatin. However, sildenafil was shown to improve kidney function and attenuate experimental cisplatin-induced nephrotoxicity [11–28,40,41]. Also gemfibrozil was shown to attenuate cisplatin nephrotoxicity either alone or in combination with silymarin [27]. It was shown that cisplatin deactivates PPAR α by reducing its DNA binding activity and the availability of its tissue specific coactivator peroxisome proliferator activated receptor-gamma-coactivator-1 (PGC-1) [42]. PPAR α ligands were found to be protective against cisplatin-induced nephrotoxicity [43].

Linear regression analysis among control and cisplatin nephrotoxicity group was done to detect the independent predictor for change in serum creatinine. HO-1 was found to be the independent predictor for serum creatinine changes and nephrotoxicity. We again did the linear regression analysis among the cisplatin nephrotoxicity group and group treated with combination therapy postcisplatin to detect the independent predictor for improvement of serum creatinine. Again improved HO-1 was found to be the independent predictor for improvement of serum creatinine. Agarwal et al. were the first to demonstrate the protective role of heme oxygenase against cisplatin nephropathy [44]. Shiraishi et al demonstrated that cisplatin nephrotoxicity was significantly greater in HO-1-knockout mice [45]. HO-1 has a nephroprotective effect including antioxidant, anti-inflammatory and prosurvival properties as demonstrated by Bolisetty et al. who manipulated HO-1 gene (deletion and overexpression) using cre-lox technology and concluded that HO-1 deletion, specifically in the proximal tubules aggravated structural and functional damage during cisplatin nephrotoxicity, however, HO-1 overexpression was found to be protective [46]. Activation of PPAR α will activate HO-1 and was found to attenuate renal ischemia reperfusion injury [47]. Sildenafil was found to activate anti-oxidant genes including HO-1 and attenuate proinflammatory cytokines like TNF α and protect against renal ischemia reperfusion injury [48]. Maximum activation of HO-1 by such combination may be attributed to the excellent results obtained. Upregulation of HO-1 was found to be protective against cisplatin and contrast induced nephropathy [6,49]. The heme oxygenase system represents a promising avenue of investigation that may lead to targeted therapeutics in kidney diseases [50]. This work has the advantage of being the first to assess such combination of sildenafil and gemfibrozil either before or after cisplatin nephrotoxicity to assess for their prophylactic versus therapeutic role. Also we investigated many tissue markers for their possible role in cisplatin nephrotoxicity and determined the independent predictor of nephrotoxicity among these markers. This study has a limitation that we did not assess the effect of combination therapy pre and post in a group of animals.

## Conclusions

Combination therapy of sildenafil and gemfibrozil can be used in treatment of cisplatin-induced nephrotoxicity. If combination therapy is recommended as prophylaxis of cisplatin-induced nephrotoxicity, it is advised to be continued post-treatment and not to be used pretreatment only. Heme oxygenase system is a promising target for cisplatin-induced nephrotoxicity.
